# Angiogenesis in the New Zealand obese mouse model fed with high fat diet

**DOI:** 10.1186/1476-511X-8-13

**Published:** 2009-04-06

**Authors:** Adriana Balwierz, Anna Polus, Urszula Razny, Lukasz Wator, Grzegorz Dyduch, Romana Tomaszewska, Stephan Scherneck, Hans Joost, Aldona Dembinska-Kiec

**Affiliations:** 1 Department of Clinical Biochemistry, Collegium Medicum, Jagiellonian University, Cracow, Poland; 2 Postgraduate School of Molecular Medicine, 61 Żwirki i Wigury Str., 02-091 Warsaw, Poland; 3 Department of Pathomorphology, Collegium Medicum, Jagiellonian University, Cracow, Poland; 4 German Institute of Human Nutrition, Potsdam-Rehbrücke, Germany

## Abstract

**Background:**

Obesity and its complications lead to vascular injury, atherosclerosis, diabetes and pathological angiogenesis. One of the models to study the obesity and its entanglements is the New Zealand Obese mice model. Aim of this study was to check the effect of high fat diet on changes in biochemical parameters as well as on process of angiogenesis in NZO mice.

**Methods:**

NZO mice were fed with standard (ST) or high fat (HF) diet for seven weeks. Body weight and serum biochemical parameters were monitored. The PECAM1 positive vessel-like structures immunostaining, as well as the gene expression of the matrigel penetrating cells by microarray (confirmed by real-time PCR method) were analyzed.

**Results:**

Mice fed with HF diet developed obesity. Number of newly created vessels with lumen was correlated with hyperglycemia and animal weight gain. The number of PECAM1 positive cells in matrigel tended to increase during HF diet. Microarray results revealed changes in gene expression (activation of the oxidative stress and insulin resistance, inhibition of apoptosis and cell differentiation), however without markers of endothelial cell network maturation.

**Conclusion:**

Observed changes in the NZO mice on HF diet argue for the hyperglycemia related activation of angiogenesis, leading to the formation of pathological, immature network.

## Introduction

Obesity, insulin resistance (hyperinsulinemia, hyperglycemia), dyslipidemia (hypertriglyceridemia with low blood HDL levels) hyperleptinemia and hypoadiponectinemia, elevated biochemical parameters of inflammatory response, as well as activation of coagulation cascade are main features of metabolic syndrome leading to micro- and macrovascular injury, hypertension, and atherosclerosis [1,2]. These events impair vessel functions and lead to diabetic retinopathy, which is a major cause of blindness in industrialized countries [3].

High fat (HF) diet promotes progression of impaired glucose tolerance, induces insulin resistance and with chronic inflammation lead to endothelial dysfunction. Vision-threatening complication of diabetic retinopathy is characterized by development of retinal neovascularization and loss of vision [4]. Local tissue ischemia, due to endothelial dysfunction, increases the production of proangiogenic substances such as vascular endothelial growth factor (VEGF), angiogenin, VEGF receptor-2 (KDR), paralleled by decrease of the inhibitors of angiogenesis such as Pigment Epithelium Derived Factor (PEDF) result in retinal neovascularization as well as microangiopathy [5,6]. VEGF has been shown to stimulate generation of nitric oxide (NO) in endothelial cells [7]. NO is known to modulate vascular permeability, dilate vasculature, and alter the transmural pressure [8]. Both ischemia as well as VEGF was found to mobilize bone marrow for the release of the endothelial progenitors which participate in the angiogenesis as well as in the pathological vessel wall remodeling [9]. Hyperglycemia stimulates the transforming growth factor β (TGF-β) expression in endothelium and vascular smooth muscle cells [10,11]. Present in circulation free fatty acids (FFA) are toxic to endothelial cell as well as to pancreatic β-cells [12,13].

Hyperinsulinemia stimulates endothelium and vascular smooth muscle proliferation, causes vasoconstriction and increase of adrenergic system tonus, thus also leads to pathological angiogenesis and vessel wall remodeling [14]. Leptin was demonstrated to stimulate angiogenesis through expression of the VEGF receptors [15], and promotion of the progenitor cell differentiation [16]. Adiponectin induces NO release and acts protectively to vessels endothelium [17,18]. Thus, the decrease of adiponectin level, associated with obesity, also contributes to endothelial dysfunction in metabolic syndrome [19].

New Zealand Obese (NZO) mice exhibit polygenic syndrome of hyperphagia, obesity, insulin resistance hypercholesterolemia [20,21]. HF diet markedly enhances development of diabetes in this mouse [22]. Recently it has been reported by our group, that increased hyperleptinemia in obesity could modify the angiogenic effect in NZO mice [23]. Moreover this syndrome is accompanied by a marked elevation of leptin in adipose tissue and serum [22], whereas adipose tissue mass grown is trongly associated with angiogenesis process [23].

In this study the NZO mice model was implemented to investigate the correlation between main biochemical parameters and the pathological angiogenesis after feeding mice with high fat diet.

## Methods

### Mice

Study was approved by the University Ethic Committee (No 58/OP/2003) and performed in accordance with the policies regarding the human care and use of laboratory animals.

NZO mice (NZO/H1Bom) were obtained from Institute of Human Nutrition (Potsdam-Rehbrucke, Germany). All animals up to 6 weeks were fed with a standard laboratory diet, had free access to food and water, and were housed in cages (22°C temperature and 12 hour daylight cycle). Six weeks old female NZO mice (n = 21 animals) were used for the experiment. The control group of NZO mice were fed ad libitum with standard-control diet (n = 11). The investigated group of NZO female mice (n = 10) were put on HF diet for seven weeks with free access to water. The standard rodent diet contained 3% fat, while 39% of total energy of the HF diet was derived from coconut oil (SFA) (MP Biomedicals, Livermore, CA, USA). During the time of experiment body weight was measured 3 times a week and chow uptake was monitored. Mice were killed at the age of 13 week by thiopental anesthesia.

### Biochemical parameters

Blood samples were collected weekly from the tail vein, after 4 hours of fasting. Serum concentrations of glucose, triglycerides and total cholesterol were monitored weekly (Cormay Diagnostic Kit, Lublin, Poland). Insulin, leptin and adiponectin concentrations were estimated in serum of the thirteen week old mice at the end of experiment period with use of the kits (Linco Research, St. Charles MO, USA and R&D Systems, Minneapolis, MN, USA). The fluctuated blood level of glucose, cholesterol and triglycerides were expressed as the area under curve (AUC) calculated by Trapezoidal Rule [24].

### The matrigel model of angiogenesis [25]

One week before the end of feeding period, mice received subcutaneous injections containing 25 nmol/L bFGF (basic fibroblast growth factor) (Sigma-Aldrich, St. Louis, MO, USA) matrigel basement (Becton Dickinson, Franklin Lakes, NJ, USA). After euthanizing mice matrigel plugs were removed and preserved for immunostaining as well as for analysis of gene expression.

Excised with surrounding tissue matrigel plugs were used for immunohistochemical staining with rat anti-mouse CD31 (PECAM1) antibody (Becton Dickinson) [26].

Angiogenic response was estimated by the amount of PECAM1-positive structures and expressed as the number of vessels with or without the lumen as well as number of individual PECAM1-positive cells counted with a Hot Spots method in 5 frames per slide and 3 slides per plug by the not informed pathologist.

### RNA isolation and gene expression analysis

To investigate the influence of HF diet in comparison to standard diet on gene expression in cells migrated to injected matrigel plug, relative gene expression was screened using the microarray assay and real-time PCR.

RNA for gene expression analysis was isolated from matrigel plugs excised without surrounding tissue. Affymetrix 430A_2 GeneChips were used for oligo hybridization and results were analyzed with Affymetrix Microarray Analysis Suit. Changes in relative gene expression were calculated as a rate of high fat against standard diet using GeneChip Operating Software (GCOS 1.4). Only genes with significant differences in signal intensity of at least 1.4 fold and p < 0.05 were included for further analysis. Analysis of regulated pathways was performed using Genmapp and MetaCore software.

In order to confirm expression of chosen genes previously indicated with the microarray, the Real-Time PCR was performed for RNA samples derived from infiltrating matrigel cells of animals fed with standard and high fat diet. The relative expression rates were calculated as the normalized CT difference between a control probe and a sample with the adjustment for the amplification efficiency relative to the expression level of the Gapdh reference gene. Calculation was performed using program Calculation Matrix for PCR Efficiency RAST-XL.

### Statistical analysis

Results were shown as mean value ± standard deviation (SD). For multiple records (Glucose; Cholesterol; triglycerides – TG) area under curve (AUC) was estimated. Number of CD31 (PECAM1) positive structures was normalized with a base-2 logarithm (log2). Analysis of differences was performed with the ANOVA Benferoni test for multiple comparisons. For correlation the Spearman Rank-order or Pearson's correlation was used.

## Results

### Development of obesity with hyperinsulinemia and elevated glucose, cholesterol and leptin concentrations after fed of NZO mice fed with high fat diet

NZO mice diet increased their weight up to 20 ± 1.9 g (110% of the startup mass) on HF, whereas on standard diet gained 11.3 ± 3.2 g (64% of their startup body weight). Those differences were statistically significant (Table 1). Moreover the HF diet significantly increased serum glucose and cholesterol levels as well as serum insulin and leptin concentration (Table 1) in NZO mice as compared to the control diet. All those differences were statistically significant and leptin concentration correlated (R = 0.92) with the weight gain. TG and adiponectin levels in blood serum did not differ significantly between groups of animals.

**Table 1:** Comparison of final body weight and serum parameters of NZO females between high fat diet fed animals (HF) and controls.

	Standard diet (control)	High fat diet (HF)
**End body weight (g)**	31,23 ± 2,33	38,04* ± 3,09
**Glucose (AUC)**	58.43 ± 4.39	77.73* ± 5.09
**Cholesterol (AUC)**	24.04 ± 3.82	44.87* ± 2.27
	
**Insulin [ng/ml]**	0.92 ± 0.47	2.47* ± 1.07
	
**Leptin [ng/ml]**	18.77 ± 16.24	73.93* ± 11.94

Mean ± SD; from ST (n = 11), HF (n = 10); significance *p < 0.01.

### Changes in angiogenic response in NZO mice fed with high fat diet

To evaluate effect of high fat diet on angiogenesis, the s.c. injected matrigel plug model was used. The number of CD31 positive: vessels with lumen, vessels without lumen and single CD31 positive cells were calculated.

**Figure 1:**
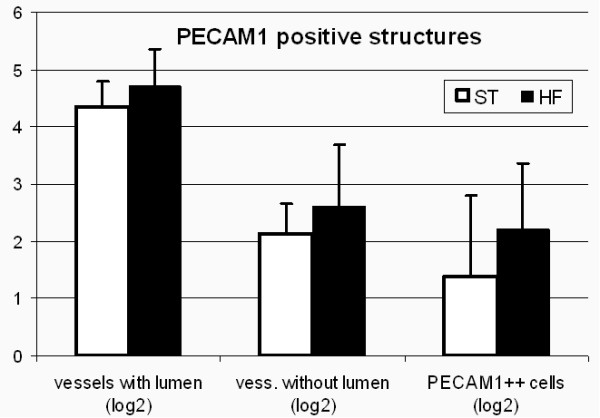
**Comparison of normalized numbers of Cd31 positive structures present in matrigel plug. Mean ± SD; from ST (n = 10), HF (n = 8)**.

The tendency for increased number of CD31 positive structures (vessels with lumen, vessels without lumen and single cells) was observed in HF group, however differences between diets were not statistically significant (Fig. 1).

**Figure 2:**
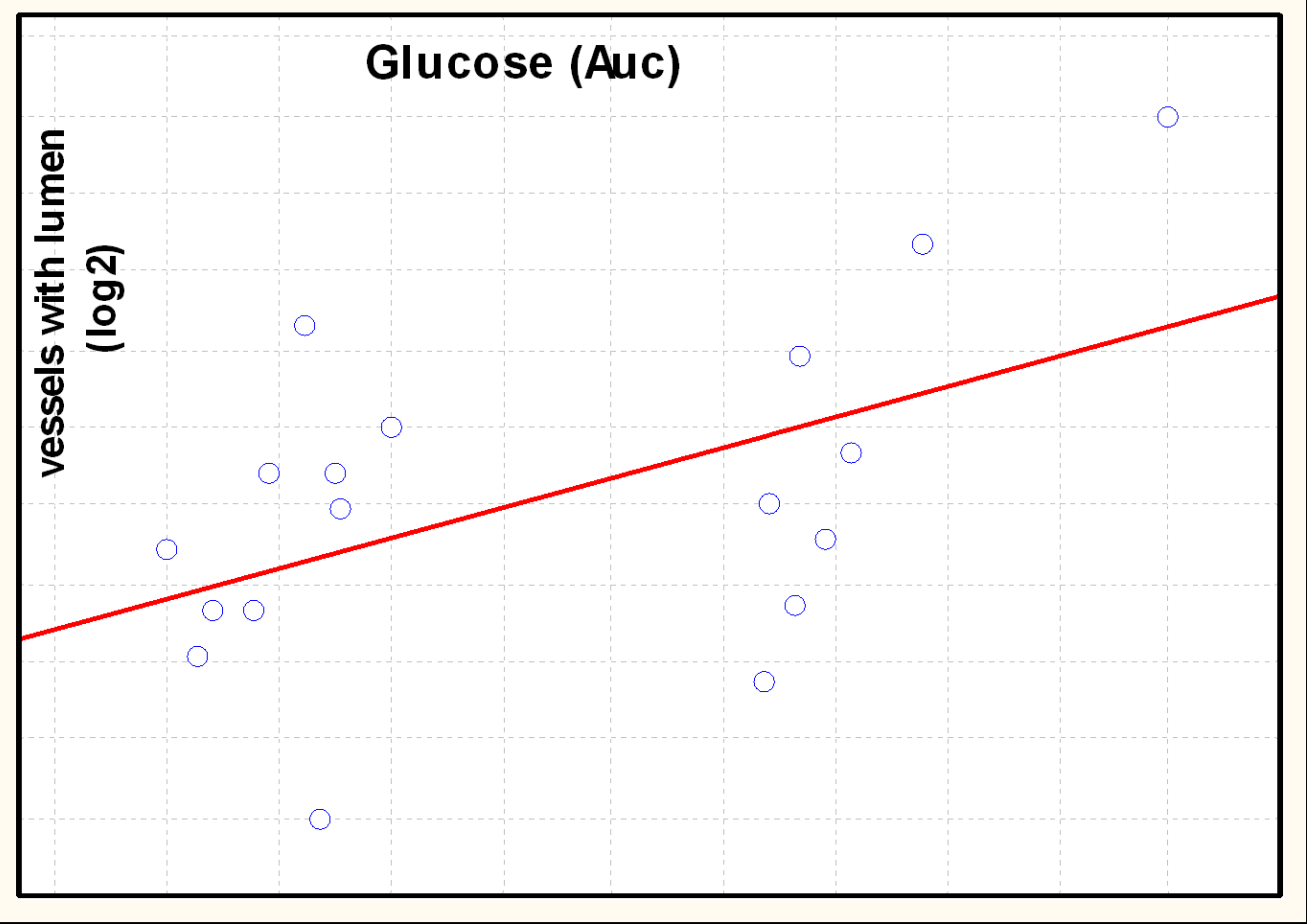
**Spearman Rank-order correlation between normalized numbers of newly created Cd31 positive structures in matrigel and area under curve of glucose for NZO mice**. R = 0.54; n = 18; p < 0.05.

The Spearman Rank-order correlation between normalized numbers of newly created CD31 positive structures in matrigel and concentration of potentially angiogenic substances such as insulin, leptin, adiponectin glucose and lipids was performed. The positive correlation between normalized (base-2 logarithm) number of vessels with lumen and area under curve of glucose was found (r = 0,54) (Fig. 2).

**Figure 3:**
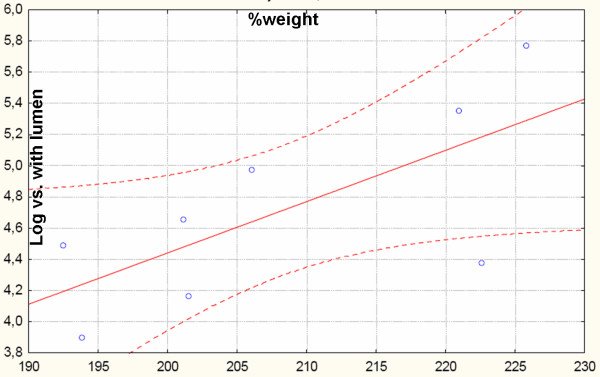
**Pearson's correlation between normalized numbers of newly created vessels with lumen in matrigel and weight gain of NZO mice on HF diet. R = 0,7; n = 8; p < 0.05**.

The positive correlation was also observed between weight gain and normalized numbers of vessels with lumen in mice fed with the HF diet (Fig. 3). Weight gain was calculated as a percentage of differences between end point weight and startup weight divided by startup weight. No such correlation was observed in mice fed on standard diet (data not shown).

### Analysis of changes in gene expression by microarray as well as real-time PCR

To check, if the observed tendency for increased number of CD31 positive structures in matrigel plugs in response to HF diet could point the changes in angiogenesis process, the microarray analysis as well as real-time PCR of cells infiltrating matrigel plugs were performed (Table 2).

**Table 2:** Relative gene expression [HF vs. ST] – microarray data

**Inslulin signaling**		**Proangiogenic cytokines**		**Adhesion molecules**	
↑ *Igf1r*	1,41	↑ *Vegfa*	9,19 ‡	↑ *Itga5*	4,92
↓ *Irs1*	-1,62	↑ *Egf*	1,87	↑ *Itga6*	1,62
↓ *Pik3r1*	-1,62	↑ *Fgf1*	2,00	↑ *Itgam*	4,59
↓ *Pik3r2*	-1,41	↑ *Fgf18*	3,48	↑ *Itgb2*	2,46
↓ *Akt2*	-1,52	↑ *Hbegf*	7,46	↑ *Itgb1*	2,14
↓ *Foxo1*	-1,87 ‡	↑ *Pdgfa*	3,25	↑ *Gja1*	1,62
				
↓ *Foxo3a*	-1,52	**Proangiogenic receptors**		↓ *Pcdhb7*	-2,83
↓ *Glut4*	-2,64 ‡	↓ *Kdr*	-8,00	↓ *Icam1*	-2,00
				
**mTor**		↓ *Flt1*	-4,29	↓ *Icam2*	-13,00
↑ *Frap1*	1,41	↓ *Egfr*	-2,83	↓ *Vcam1*	-6,06
↑ *Rheb*	1,52	↓ *Tie1*	-10,56	↓ *Mcam*	-19,70
↑ *Tsc2*	1,74	*Fgfr1*	1,32	↓ *Pecam1*	-6,96
			
**Cell cycle**		↓ *Cxcr4*	-2,30	**Extracellular matrix**	
				
↓ *Ccna2*	-1,62	**Sphingosine 1-P**		↑ *Lamb1-1*	2,83
↓ *Cdc2a*	-1,41	↑ *Sphk1*	1,62	↑ *Fn1*	1,41
↓ *Cdc20*	-1,52	↑ *Edg1*	2,46	↓ *Col18a1*	-3,48
				
↓ *Mcm3*	-1,87	**Proang. trans. factors**		↓ *Col1a1*	-1,74
↓ *Mcm5*	-1,87	↓ *Hoxd9*	-34,30	↓ *Col1a2*	-1,62
↓ *Mcm7*	-1,41	↓ *Hoxd10*	-7,46	↓ *Col4a1*	-7,46
↓ *Orc2l*	-2,83	↓ *HoxA9*	-4,29	↓ *Col4a2*	-6,50
				
**Cell cycle inhibitors**		↓ *HoxA10*	-8,00	↓ *Col4a5*	-2,46
↑ *Akt1*	1,87	↑ *Jun*	1,62	↓ *Col6a1*	-2,64
↑ *p53*	1,62	↑ *Fos*	6,06	↓ *Col6a2*	-3,25
↑ *p21*	1,87	↑ *Egr1*	2,14	↓ *Fgg*	-3,73
				
↑ *p16*	2,83	**Diff. EC markers**		↓ *Sparc*	-1,41
↑ *Ywhag*	2,46	↓ *vWf*	-4,29	↓ *Sparcl1*	-16,00
				
**Oxidative stress**		↓ *Esam1*	-10,56	↓ *Smoc2*	-4,59
				
↑ *Hspa1a*	6,50	↓ *eNos*	-2,83 ‡	**MMP's & MMP likes**	
				
↑ *Hspb1*	2,46	**Apoptosis**		↑ *Mmp1a*	2,46
↑ *Hmox1*	2,30	↓ *Bak1*	-1,87	↑ *Mmp10*	9,19
↑ *Ankrd1*	6,06	↓ *Bad*	-1,74	↑ *Mmp14*	1,87
				
**Prostaglandin activity**		↓ *Bcl2L11*	-3,03	↑ *Mmp19*	2,00
↑ *Cox1*	2,46	↓ *Casp12*	-1,87	↑ *Adam8*	2,14
↑ *Cox2*	6,50	↓ *Casp7*	-1,62	↑ *Adam17*	1,41
↑ *Ptgis*	2,00	↓ *Prf1*	-4,92	↑ *Adam19*	1,87
↑ *Ptgir*	2,46	↓ *Gzmb*	-5,66	↑ *Adamts4*	2,30
				
**Plasminogen act.**		↓ *Birc2*	-2,00	↓ *Mmp12*	-1,62
↑ *Serpine1*	7,46	↓ *Birc5*	-2,00	↓ *Mmp9*	-1,87
				
↑ *Serpinb2*	29,86	**Integrin related kinase**		↓ *Mmp2*	-1,62
↑ *Plau*	3,25	↑ *Rock1*	1,74	↓ *Mmp23*	-2,14
↑ *Plaur*	4,00	*Rock2*	1,23	↓ *Adamts15*	-7,46
				
↑ *Ilk*	1,41	↑ *Rac1*	1,41	**Colour explanation:**	
↑ *Crk*	1,87	↑ *Pak1*	1,52	up-regulation ↑	
				
↑ *Rapgef1*	2,83			down-regulation ↓	
↑ *Rap1b*	2,14				

‡ regulation confirmed with Real time PCR

The analysis of microarray results from matrigel cells identified the 3719 regulated genes, which expression was changed in response to the HF diet (1658 genes were up-regulated, while 2061 were down-regulated).

In response to HF diet stimulation insulin signal transduction was suppressed due to down-regulation of Pik3r1, Pik3r2, and Akt2 genes. Mammalian Tor (mTOR) pathway was activated, confirmed by Frap1, Rheb and Akt1 up-regulation. This resulted in the insulin resistance marked by the down-regulation of insulin receptor signalling (Irs1, Foxo1, and Foxo3a), and down-regulation of Glut4.

Prostaglandin synthases (Cox1, Cox2, Ptgis) as well as prostaglandin receptor (Ptgir) gene expression was increased by the h HF diet.

High fat diet activated important for cell cycle cyclin-dependent kinase inhibitors p53, p21, p16, and Ywhag. Also expression of proteins which play crucial role for G1/S and G2/M transitions such as: Cyclin A2 (Ccna2), and activated by this cyclin Cdc2a, Cdc20 as well as genes (Mcm3, Mcm5, Mcm7, Orc2l) encoding proteins involved in the initiation of DNA replication was down-regulated in matrigel cells from high fat fed mice.

Genes participating in oxidative stress: heat shock proteins 70 (Hspa1, Hspb1), heme oxygenase (Hmox1) were up-regulated in HF group.

High fat diet up-regulated gene expression of numerous growth factors involved in process of angiogenesis including Vegfa, epidermal grow factor (Egf), fibroblast growth factor 1 (Fgf1), fibroblast growth factor 18 (Fgf18), heparin-binding EGF-like growth factor (Hbegf), platelet derived growth factor a (Pdgfa), however their receptors (Kdr, Flt1 Egfr, Tie1) gene expression was inhibited.

Adhesion dependent activity of cells such as migration, proliferation, cell survival, and matrix degradation was proved to contribute to angiogenic process [27,28]. HF diet activated expression of genes of endothelium integrins (Itga5, Itga6, Itgam, Itgb1, Itgb2) which participate in cell-matrix interactions. It was associated with the up-regulation of genes, which encode proteins taking part in cell-cell interaction such as cadherins (Ve-cadherin: Pcdhb7), and gap junction protein (Gja1). Adhesion molecules contributing to endothelium-leucocytes interactions (Icam1, Icam2, Vcam1, Mcam, Pecam1) were down-regulated by HF diet. Contributing to capillary tube formation, extracellular matrix proteins laminin-1 (Lamb1-1) and fibronectin (Fn1) ligands for endothelial integrin genes were up regulated by high fat diet. Genes encoding matrix protein such as collagen and fibrinogen (Fgg) were down-regulated. Also genes of Sparc (Sparc, Sparcl1, Smoc2), which is the matrix-associated protein able to inhibit cell-cycle progression, and influence matrix extracellular synthesis [29] were inhibited by HF diet.

Endothelial cell apoptosis plays a critical role in physiologic and pathological vascular regression remodeling [4] and angiogenesis [27,28]. In cells isolated from matrigel, HF diet down-regulated expression of pro-apoptotic Bcl-2 family members: Bak1, Bad, and Bcl2L11. Genes important for sequential activation of caspases cascade Casp12, Casp7, Prf1, Gzmb were also down-regulated in mice fed with HF diet, however the apoptosis inhibitor proteins Birc2, Birc5 were suppressed. Expressions of importance for TNF-α signal transduction pathway proteins were also inhibited by HF diet.

Genes participating in oxidative stress: heat shock proteins 70 (Hspa1, Hspb1), heme oxygenase (Hmox1) were up-regulated in HF group.

In order to confirm microarray results, the expression of genes taking part in processes which may contribute angiogenic response and glucose metabolism was verified with the real-time PCR. Up-regulation by the high fat diet, has been confirmed for genes encoding: Vegfa, and Trp53. Down-regulation has been confirmed for genes encoding: eNos, Pecam1, Kdr, Glut4, and Foxo1. According to real-time PCR data, genes encoding: connexin 43 (Gja1), and Frap1 was found not to be regulated by diet (Table 2).

## Discussion

The NZO mice model has been recommended for study obesity and related complications, because it has a polygenic syndrome that resembles human metabolic syndrome, with hyperphagia, obesity and insulin resistance [22]. An effect of high fat diet to induce growing of fat cell size, that lead to development of obesity in these animals is also known [29]. The purpose of presented work was to evaluate the effect of high fat diet on angiogenesis process in the New Zealand Obese (NZO) mice.

Elevated concentrations of glucose, insulin, cholesterol, and leptin in blood serum of mice fed with HF diet, observed in our experiments, could provide multiple signals to the vessel wall. HF diet promotes angiogenesis in NZO mice, measured by the increased number of PECAM1 positive cells migrating into matrigel and forming primitive tubular-like structure. The angiogenic potential of cells migrating to matrigel in murine model of angiogenesis was presented for murine [25] as well as for human progenitor cells [30]. Such process could be linked to Akt signaling [31]. The down-regulation of Akt gene in microarray results points to lack of maturation of cell migrating to matrigel, since in Akt-defficient mice the enhance of angiogenic response with impairment of blood vessels maturation has been found by others [31].

Adipose tissue, stimulated by insulin, is a source of proangiogenic factors, such as VEGF, Il-8, and Serpin1 [32]. Thus, growing mass of adipose tissue provides signals for angiogenesis. Microarray results from cells invading matrigel are consist with above suggestion, since high fat diet stimulated the gene expression of numerous proangiogenic factors like Vegf, Egf, Fgf1, Hbegf, Pdgfa, Fgf18. However the gene expression of some cytokine receptors was suppressed, whereas the others suggest, that insulin and/or glucose stimuli might up-regulate the Vegf gene expression in NZO mice fed with high fat diet [33]. Insulin may act on angiogenesis through regulating several pathways. Nitric oxide (NO) release is one of the possibility with the major impact. However, the microarray results indicate that the downstream insulin receptor, as well as expression of endothelial NO synthase (eNos) was suppressed by the HF diet, what agree with the recent knowledge [34]. Elevated concentration of glucose and leptin increase respectively cyclooxygenase-2 (Cox2) and cyclooxygenase-1 (Cox1) gene expression promoting synthesis of prostacyclin and prostaglandins with protective activity on endothelium [35]. In group of mice fed with high fat diet, Cox1, Cox2 genes as well prostaglandin synthase and prostacyclin receptor expression were activated, what may lead to synthesis of pro-inflammatory prostanoids [35].

It has been demonstrated that also leptin contributes to angiogenesis [36]. Leptin has been shown to cause aggregation, formation of tubes and disposing of tissue vasculature of cultured endothelial cells [25]. In study on NZO mice, obesity correlated with leptin concentration, and the group fed with HF diet, demonstrated hyperleptinemia. However neither leptin nor adiponectin concentrations correlated with the angiogenic response, nor find confirmation in microarray results showing down-regulation of the leptin signaling by JAK-STAT pathway in animals on HF diet.

Among investigated parameters serum glucose concentration correlated positively with the number of matrigel vessels with lumen. Excessive exposure to glucose may cause multiple actions on endothelial cells. The generation of reactive oxygen species (ROS) is mechanism responsible for the majority of the glucose effects such as activation of NF-kappaB or protein kinase C (PKC) [37,38]. Up-regulation of AP-1, SMAD, heme oxygenase (Xmox1) and heat shock proteins is a strong evidence for ROS generation in hyperglycemic NZO mice [39]. Reactive oxygen species might activate SMAD, AP-1 transcription factors and trigger biglycan, integrin and metalloproteinases synthesis, contributing to cell adhesion, migration, and extracelluar matrix breakdown thus promotion of angiogenesis [40].

Indirectly ROS are also a cause of intensive protein synthesis which leads, either proliferation or apoptosis. This effect may be related to increased angiogenesis, since Akt1 which acts for cell survival and protein synthesis was up-regulated by HF diet. Activation of the mammalian target of rapamicin (mTOR) signaling observed in microarray confirms the increase in protein synthesis in HF NZO. Additionally acts for increase of VEGF expression (through HIF-1α activation) and previously mentioned insulin resistance observed in these animals.

Observed on HF diet up-regulation of stromelysins (Mmp3, Mmp10) is characteristic for wound healing process. Additionally inhibition of apoptosis may explain the growing number of cells in matrigel without activation of proliferation. Together, with endothelial adhesion molecules and extracellular matrix proteins, metalloproteinase provide signal for weakened cell-cell interaction, which can cause vessel wall permeability, endothelial cell migration and tubule formation [41]. It may lead to pathological angiogenesis and outgrowth of vessels without markers for mature endothelium (vWf, Esam1, Pecam1, eNos).

The process of adipose tissue mass growth is highly coupled with angiogenesis, adipogenesis as well as extracellular matrix remodeling, and blood vessel density in adipose tissue can be normalized to the adipocyte density [25]. This information is in agreement with our findings, where the positive correlation, for weight gain of mice fed on HF diet and the number of vessels with lumen was found. Together with microarray results this could indicate, that high fat diet induce the formation of premature vessels.

The high fat diet enhanced symptoms of metabolic syndrome in the NZO mice, such as obesity, hyperinsulinemia, hyperglycemia, hypercholesterolemia, and hyperleptinemia. According to microarray, the activation of early stages of angiogenesis (migration, inhibition of apoptosis, changes in adhesion and matrix remodeling), but not the endothelial cell differentiation was stimulated and is suggested to be mainly dependent on glucose level due to the positive correlation between blood glucose and immature angiogenic response.

## Competing interests

The authors declare that they have no competing interests.

## Authors' contributions

AB carried out the molecular biology studies and drafted the manuscript. AP planned all experiments and look after the progression of study. UR participated in the animal studies as well as biochemical analysis. LW participated in the animal studies, biochemical and statistical analysis as well as he drafted the manuscript. GD prepared immunohistochemical staining matrigel plugs. RT prepared immunohistochemical staining matrigel plugs. SS design the NZO mice (NZO/H1Bom) model. HJ design the NZO mice (NZO/H1Bom) model. ADK planned the study, and participated in its design and coordination as well as prepared the manuscript. All authors read and approved the final manuscript.
